# Identification of miR-328-3p as an endogenous reference gene for the normalization of miRNA expression data from patients with Diabetic Retinopathy

**DOI:** 10.1038/s41598-019-56172-w

**Published:** 2019-12-23

**Authors:** Marcelle SanJuan Ganem Prado, Thaline Cunha de Goes, Mirthz Lemos de Jesus, Lucilla Silva Oliveira Mendonça, Jadson Santos Nascimento, Carla Martins Kaneto

**Affiliations:** 1 Department of Health Science, Universidade Estadual de Santa Cruz, Ilhéus, BA Brazil; 2 Department of Biological Science, Universidade Estadual de Santa Cruz, Ilhéus, BA Brazil

**Keywords:** Gene expression, Diagnostic markers, Retinal diseases, Molecular medicine

## Abstract

Diabetic Retinopathy, the main cause of visual loss and blindness among working population, is a complication of Diabetes mellitus (DM), which has been described as a major public health challenge, so it is important to identify biomarkers to predict and to stratify patient´s possibility for developing DR. MicroRNAs (miRNAs) are small non-coding RNA molecules that have showed to be promising disease biomarkers and association of miRNAs with the possibility to develop DR has been reported. However, evaluating miRNA expression involves normalization of RT-qPCR data using internal reference genes that should be properly determined, considering their impact on expression levels calculation and, until date, there is no unanimity on reference miRNAs for the investigation of circulating miRNAs in DR. We aimed to estimate the appropriateness of a group of miRNAs as normalizers to identify which might be considered steady internal reference genes in expression studies on DR plasma samples. Expression levels of candidates were analyzed in 60 healthy controls, 48 DM without DR patients and 62 DR patients with two statistical tools: NormFinder and RefFinder. MiR-328-3p was the most stable gene and we also investigated the effect of gene normalization, demonstrating that different normalization strategies have important implications for accurate data interpretation.

## Introduction

Diabetic retinopathy (DR), the main cause of vision loss and blindness in adults, is the most frequent microvascular complication of diabetes mellitus (DM)^1,2^. The main reasons for this decline in visual acuity are blood-retinal barrier disruption with consecutive diabetic macular edema and retinal angiogenesis, but the exact mechanism that leads to vascular disruption is not defined^3^. Longer duration of DM and poor blood glucose control constitute main risk factors for DR, but a considerable number of patients that do not present these risk factors develop DR while there are patients with long DM duration that do not progress to this complication^4^. Usually, it is not easy to infer about the clinical course of the disease and, besides this, many patients do not take or have a good response to the stablished primary and secondary therapy for DR, which includes controlling risk factors, medication and advanced treatments, like ocular laser, anti-vascular endothelial growth factor medicine and corticosteroids, emphasizing the necessity of development of highly sensitive, specific and widely available clinical laboratory-based monitoring tests for this condition and the relevance of improving our knowledge about the characteristics of DR^5^. Incorporating biomarker analysis into determining the risk of people with DM would allow a faster diagnosis and would also help in the choice of a better treatment for those with DR. A biomarker can be described as a measurable substance that could be evaluated as a sign of physiological and pathogenic processes or response to a medical treatment and the measurement of such biological markers must be applicable for clinical decision making.

Circulating microRNAs (miRNAs) have been currently described as good non-invasive biological markers for diagnostics, disease stratification and prognostic determination. MicroRNAs are single stranded, non-coding RNA molecules with short length (22 to 26 nucleotides) that are able to bind to the complementary sites of target mRNAs, controlling gene expression in a post-transcriptional manner, presenting crucial functions in gene regulatory networks^6–8^. Recently, miRNAs were found to be promptly liberated from tissues into the blood as a pathology develops and atypical expression of circulating miRNAs has been observed in a large number of pathologies such cancer^9,10^, diabetes^11,12^, cardiovascular^13–15^ and neurodegenerative^16^ diseases. This cell-free molecules were also found circulate in a stable manner in many body fluids such as serum^17,18^, plasma^19,20^, saliva^21^, urina^22^ and milk^23^ and these observations have encourage the utilization of extracellular circulating miRNAs as non-invasive biological markers for molecular diagnostics and prognostics.

Reverse transcription quantitative polymerase chain reaction (RT-qPCR) has been described as a precise method for circulating miRNA detection and quantification. Despite this, meticulous quantification of miRNA levels is necessary because discrepancies in the quality and quantity of starting material, sample collection and storage, RNA extraction process, RT (reverse transcriptase) and PCR (Polymerase Chain) reactions performance may cause potential bias and generate quantification errors, affecting their use as molecular markers and the determination of the differentially expressed miRNAs functions. Considering all these factors, the development of an efficient normalization protocol, which could reduce errors in the miRNA expression quantitation and technical variation in experiment, is essential for estimating circulating miRNAs expression and the appropriated choice of this normalization strategy plays a central role that needs to be considered. Among diverse described normalization strategies including the mean global expression^24^ and exogenous spike-in artificial synthetic oligonucleotide^25^, normalization using a stable internal, reference or endogenous gene^26^ (or a group of multiple reference genes with stable expression^27^) is the most rigorous and commonly used method for the determination of miRNA expression levels at present. However, until now, there is no unanimity on reference miRNAs for the normalization of circulating miRNAs in RT-qPCR amplification, especially because their expression is highly affected by the pathology, so it would not be possible to find a unique reference miRNA which could be suitable for all types of samples and diseases. So, a careful validation of the internal reference genes should be conducted for different study populations, samples and diseases in order to obtain reliable data about miRNA expression^28^. Until date, there is no consensus on internal reference miRNAs for RT-qPCR studies of circulating miRNAs in DR.

In this work, we investigated candidate miRNA reference genes in DR aiming to stablish miRNAs that could be used as good and reproducible internal reference for plasma miRNAs quantification in healthy people, DM without DR patients and DR patients to allow further application in miRNA expression studies. Initially, 10 miRNAs were elected as internal reference gene candidates to normalize RT-qPCR data. Secondly, the expression stability of 4 candidate genes was evaluated using two statistical tools: RefFinder^29^ and NormFinder^30^. Finally, we evaluated the consequence of using different combinations of reference genes on target miRNA expression.

## Results

### Selection of candidate reference miRNAs and stability analysis

Initially, the expression of 10 miRNAs (RNU6B, U6snRNA, MiR-29a, MiR-99a, MiR-27b, MiR-328-3p, MiR-342-3p, MiR-320a, MiR-155 and MiR-34a) was evaluated in plasma samples of 10 subjects of each group. For RNU6B, considered a reliable normalizer for intracellular and circulating miRNA expression analysis, miR-27b, miR-155 and miR-34a we could not detect any expression. All them presented raw Ct values ≥ 39 (data not shown). MiR-29a and MiR-99a also showed a low expression and homogeneity over the samples, with median Ct values of 37.3 and 38.2, respectively. The expression levels of the four remaining candidates (U6snRNA, MiR-320a, MiR-328-3p and MiR-342-3p) were compared in the remaining 50 healthy controls, 38 DM without DR patients and 52 DR patients to validate candidate endogenous reference genes.

**Figure 1:**
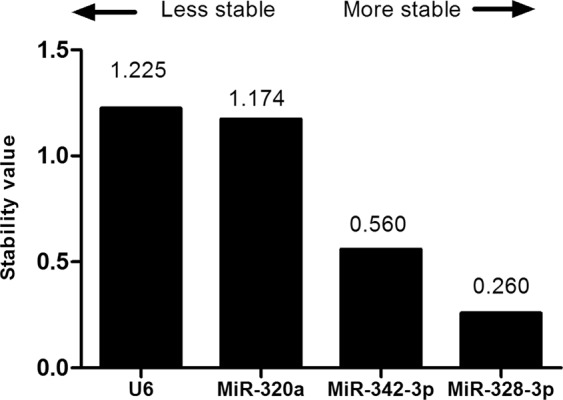
Ranking of the candidate endogenous normalizers using NormFinder. The expression stability of four candidate endogenous normalizers was evaluated by the stabilility values that NormFinder generated by comparing plasma samples from DR, DM without DR and healthy control groups: the lower the stability value, the more stable the candidate endogenous normalize. MiR-328-3p is the most stable candidate normalizer within the data set.

All data set of input RNA and inter-run calibrator-normalized Ct-values was analyzed with the NormFinder software to perform stability analysis, estimating the values for the intra and inter-group variability. This test indicated that, among the 4 candidate genes, miR-328-3p presented the most stable expression (Stability value = 0.260) (Fig. 1). The best combination recommended by NormFinder was a group of miR-328-3p and MiR-342-3p (Stability value = 0.398).

**Figure 2:**
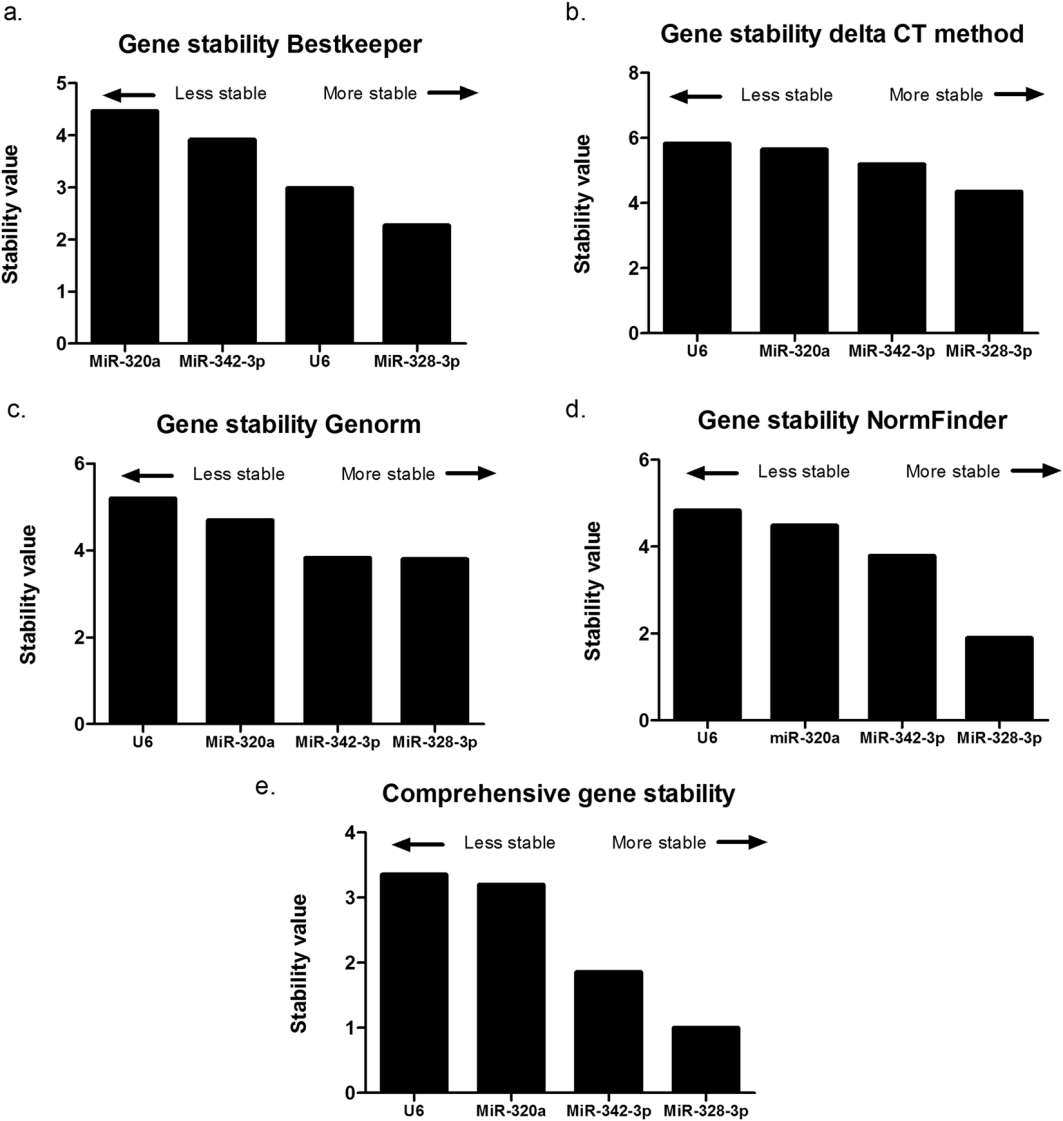
Stability of normalization candidate mature miRNAs determined with different algorithms. Stability values were calculated by the online available tools BestKeeper, Delta Ct, Genorm and NormFinder. The lower stability value, the higher stability. Each tool reveals MiR-328-3p as the most stable one as also stated by the comprehensive ranking.

Additionally, the data set was analyzed with RefFinder. This tool conjugates the normalization determination algorithms GeNorm, BestKeeper, DeltaCt and NormFinder. Stability analysis conducted with BestKeeper revealed that miR-328-3p presented the most stable expression in the group (Fig. 2a). MiR-328-3p was followed by U6 as the second stable candidate, followed by MiR-342-3p and MiR-320a. The comparative Delta Ct method (Fig. 2b) revealed the same results as the NormFinder software (Fig. 2c) or the Genorm analysis (Fig. 2d) that indicated miR-328-3p as the most stable normalization candidate, followed by MiR-342-3p, MiR-320a and U6. The NormFinder results were confirmed by the online version included in RefFinder. Assembling all the algorithms together by calculating the mean rank for each of the 4 candidate internal normalizers, MiR-328-3p followed by MiR-342-3p, MiR-320a and U6 were the best (Fig. 2e).

### Expression differences between DR, DM without DR and healthy control groups

**Figure 3:**
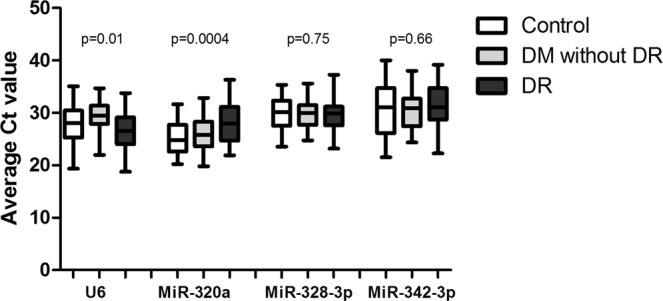
Expression differences of the normalization candidates between DR, DM without DR and healthy control patients. The expression levels of four candidate normalizers were compared between plasma of the patients from the three groups.

Since is crucial for an internal reference gene to have similar expression in diseased and healthy conditions, our data were analyzed between the three groups, looking for differences in mean Ct values. No significant differences between DR, DM without DR and healthy control groups were observed in the mean expression of MiR-328-3p and MiR-342-3p (all p-values > 0.05), but significant diffence between these groups was pointed in U6 and MiR-320a expression (Fig. 3).

### Comparative quantification of target miRNA relative to reference endogenous control gene

**Figure 4:**
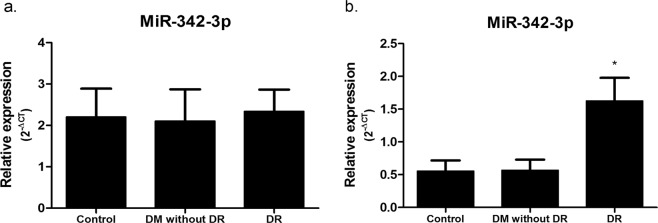
Effect of normalization on the expression of MiR-342-3p in DR, DM without DR and healthy control patients. Relative expression of DR patients (n = 62) was compared with DM without DR (n = 48) and healthy control patients (n = 60). The expression levels (2^−ΔCt^) of MiR-342-3p are presented as mean fold changes ± standard errors. Significance was calculated by one-way ANOVA test with post-hoc Tukey test. Results with p values < 0.05 were considered significant.

For normalization purposes, we tested the effect of using MiR-328-3p alone and the average of all miRNAs displaying Ct < 32 cycles as showed in previous studies^31^ on relative expression values using MiR-342-3p as target. Normalizing the expression values of MiR-342-3p to MiR-328-3p, which was shown to be the best reference gene, results in no detectable MiR-342-3p expression difference between DR, DM without DR and healthy control groups (Fig. 4a). However, the expression of MiR-342-3p normalized to the average of all miRNAs displaying Ct < 32 revelead significant upregulation in DR group (Fig. 4b), which showed difference by more than 3 times between DR, DM without DR and control groups. This analysis demonstrates that different normalization protocols significantly impact the expression quantitation results.

## Discussion

Circulating miRNAs have been described as minimally invasive molecular markers and a large number of evidences indicate they play important roles in pathological processes suggesting their use as biomarkers in several physiological and pathological conditions. qRT-PCR is an important technology and currently the most accurate method for miRNA quantitation because its sensitivity and reproducibility, but this high sensitivity requires an internal control to correct the non-biological fluctuation. An optimal internal or endogenous reference gene should have similar expression across all samples, exhibiting steady expression levels between samples and groups. It is also important that this internal control does not present association with the disease under investigation. Internal reference genes commonly used for tissue or cell miRNAs should not be used to normalize circulating miRNA expression levels because the efficiency of their isolation, reverse transcription and PCR reactions may be different of circulating miRNAs. Accurate quantitation of miRNA levels is necessary because differences in the quantity of starting material, sample collection and storage, RNA extraction process, RT and PCR performance may contribute to quantification errors, affecting their use as biomarkers. Given these concerns, these study shows the application of an effective normalization protocol, which can diminish quantitation errors and technical variability in experiment, for evaluating circulating miRNAs expression.

The mechanism by which circulating miRNAs are supposed to be protected from degradation in human body fluids suggests that these miRNAs constitute a particular group of molecules that are different from other types of RNAs in plasma and, thus circulating miRNAs themselves may be the exclusive reliable internal reference genes for normalization of circulating miRNAs. Despite this, the reference endogenous circulating miRNAs must be correctly selected and validated in order to permit precise normalization. Until now, there is no unanimity on reference miRNAs for RT-qPCR analysis of circulating miRNAs in DR.

RNU6 is a largely used reference gene in tissue and cell samples. But in our investigation, no expression could be detected as showed by raw Ct values ≥ 39, confirming recent suggestions that RNU6 was not constantly expressed in plasma and serum samples and should not be considered a reliable control for circulating miRNA expression^18,32^. U6 is another universal reference gene also used in studies investigating microRNAs expression in plasma from type 2 diabetic retinopathy subjects^33^, but our study showed a least stable expression level of U6 compared to other candidates.

In this work, we validated that miR-328-3p could be used as a suitable reference in plasma of DR patients for qRT-PCR, using the association of four statistical tools: BestKeeper, GeNorm, NormFinder and RefFinder, which were designed to determine the best internal reference gene in a given group of samples. Bestkeeper assess stability by the calculation of standard deviation and correlation of Ct values^34^, whereas geNorm considers a pairwise comparison approach presuming a similar expression ratio in all samples^35^. NormFinder enables the calculation of the stability values for a group of genes by estimating intra- and inter-group variability^30^. The overall stability ranking genes determination was conducted by the geometric mean of the rankings produced from all four analyses. The choice of the best internal reference gene for circulating miRNA expression studies in DR conducted in this study was based on the performance of the Taqman MicroRNA Assays, the quality of the related expression data and on the expression stability analysis. Using these parameters, MiR-328-3p was pointed as the best reference gene in our experimental conditions.

Many genes are frequently used as internal reference genes for tissue and cell miRNAs expression determination, but recent studies show that it is not acceptable to use these genes in the normalization of circulating miRNA levels once these genes are not miRNAs and may not represent the miRNA fraction. The use of synthetic non-human (e.g. *C. elegans*) miRNAs as spike-in controls in the normalization of circulating miRNAs, were described in previous studies^20^. This molecules are also not an a good choice once they provide a reference for normalization of the technical variance in RNA isolation, but do not correct for fluctuation in sample collection and are not able to improve assay precision^36^.

qPCR (quantitative polymerase chain reaction) data for circulating miRNAs expression can also be normalized following protocols considering total miRNA expression in the samples, proposing the use of the mean expression value of whole miRNAs in a sample to normalize miRNA qPCR data, including in DR. Zampetaki *et al*. assessed 300 patients in 2 DR-randomized clinical trials using a candidate microRNA approach, identifying miR-27b as associated with decreased risk of DR, using the average of all miRNAs displaying Ct < 32 cycles for normalization purposes^31^. The effect of these different normalization protocols was evaluated in our study. When data were normalized to MiR-328-3p, miR342-3p showed no significant difference between the three analyzed groups of patients. However, when the data were normalized to the average of all miRNAs displaying Ct < 32, significant differences were detected between DR group and DM without DR and control groups. These results demonstrate the effect of reference genes in the expression levels calculation results and the necessity of the identification of reliable internal reference genes to produce unequivocal data.

Despite the results we obtained, this study has potential limitations. Initially, the experimental context, which was specifically designed to investigate the effect of the normalization protocol, does not allow to investigate how DM duration differently influences the circulating miRNAs expression profile in DR and DM without DR groups. Secondly, this study was conducted only in a single hospital and the small sample size could difficult to identify significant relationships from the data. Besides this, only ten candidate microRNAs were selected from a review of previously published studies and based on prior related experiments. Large-scale miRNA expression studies could be more informative regarding to new candidates for miRNA expression normalization.

In summary, once an accurated validation of the internal reference genes should be conducted for different tissue and disease samples and there is no unanimity on reference miRNAs for RT-PCR analysis of plasma miRNAs in DR, our findings are the first report describing a protocol for the identification and validation of a reliable reference gene for normalization of miRNA in plasma of DR patients and that this is important for accurate experimental design and data interpretation. The results presented here suggested that the proper selection of reliable endogenous reference microRNAs is indispensable for the correct quantitation of plasma miRNA expression levels by RT-qPCR. MiR-328-3p was the most stably expressed microRNA in the present study and could be used as an optimal endogenous reference miRNA to evaluate a target miRNA expression.

## Methods

### Patients and control subjects

The subjects were recruited at CENOE (Clínica Especializada de Olhos, Ilhéus, Bahia, Brazil), all of them provided informed consent and the study was approved by the Ethical Committee of the Universidade Estadual de Santa Cruz, Ilhéus, Bahia, Brazil. All research was performed in accordance with relevant regulations and informed consent was obtained from all participants. Subjects underwent fundus flourescein angiography, which was conducted by certified ophthalmologists and were divide in three different groups: DM patients with DR (DR group), DM patients without DR (DM group) and control subjects. Inclusion criteria: DR was diagnosed according the guidelines from Global Diabetic Retinopathy Project Group^37^: after routine fundus examination and fundus fluorescence angiogryphy examination, DM patients suffering from any on of hemangioma, a few small bleeding points, neovascularization, vitreous hemorrhage or secondary retinal detachment in the retina. Exclusion criteria: patients with acute complications like diabetic ketosis, cardiovascular events, trauma opertion, acute or chronic infection, hepatic disease and other endocrine metabolic diseases. Samples with RNA 260/280 ratio lower than 1,8 were also excluded. Additionally, age, sex, BMI (body mass index), and duration of disease were recorded. The information of all donors was detailed in Table 1. Screening analysis was performed on RNA derived from blood on 10 of the above patients and controls. The remaining 50 healthy controls, 38 DM without DR patients and 52 DR patients were used to validate candidate endogenous reference genes and target miRNA expression.

**Table 1:** Clinical characteristics of the patients.

Characteristics	Control Subjects	NDR subjects	DR subjects	P value
n	60	48	62	
Age (years)	54.2 ± 13.3	58.6 ± 8.95	58.3 ± 6	0.09
Gender (male/female)	33/27	11/37	29/33	0.02*
Course of disease (years)	-	5 ± 5.2	17 ± 10.3	<0.0001*
Alcohol consumption	28	16	18	0.11
Current Smoker	10	19	23	0.01*
Kidney disease	1	-	1	0.67
Cardiovascular disease	1	1	3	0.53
BMI (kg/m^2^)	27 ± 3.5	29 ± 5.6	27 ± 5.4	0.19
**Medication**				
Biguanides (%)		50%	54%	
Sulfonylureas (%)		25%	45.1%	
SGLT2 Inhibitors (%)		2%	0	
Insuline		10%	56.4%	

*P < 0.05.

### Blood samples, RNA isolation and cDNA synthesis

Venous blood samples (5 mL) were collected from each donor in BD vacutainers containing 10 mg dipotassium EDTA anticoagulant and processed within one hour. Separation of the plasma was accomplished by centrifugation at 800 g for 10 min at room temperature to remove cell debris. Supernatant plasma was recovered and those plasma samples with pink/red discolouration were considered haemolysed and were excluded. Plasma sample of 300 µL was mixed with Trizol LS (Invitrogen) with a ratio of 1:3 in 1.5 mL microcentrifuge tube and incubated at room temperature for 5 minutes. Then, 250 µL of chloroform was added and mixed vigorously by vortex. The mixture was incubated at room temperature for 15 min and centrifuged at 12000 rpm for 20 min at 4 °C. The supernatant (approximately 400 µL) was transferred to a microcentrifuge tube. Subsequently, the RNA was precipitated by adding 800 µL isopropanol to the aqueous phase. After being incubated at −80 °C for 12 hours and centrifuged at 12000 rpm for 20 minutes at 4 °C, the RNA pellet was rinsed, air-dried and resuspended in 20 µL RNAase-free water. RNA concentrations were determined with a NanoDrop 1000 (Thermo Scientific). Only RNA samples with a 260/280 ratio of ≥ 1.8 were included. Total RNA (500 ƞg) was reverse transcribed using miR-specific primers and Taqman miRNA Reverse Transcription Kit (Applied Biosystem) in a scaled down volume of 15 µL RT reaction, according to the manufacturer’s instructions. The thermal cycling parameters of reverse transcription were 30 min at 16 °C, 30 min at 42 °C and 5 min at 85 °C. The cDNA samples were diluted in nuclease-free water and stored at −20 °C.

### Quantitative real-time PCR

Expression levels of individual miRNAs were detected by subsequent RT-qPCR using Taqman MicroRNA assays (Applied Biosystems) and a QuantStudio3 Instrument (ThermoFisher Scientific) using standard thermal cycling conditions in accordance with manufacturer recommendations. RT-qPCR amplification mixtures contained 20 ƞg template cDNA, 10 µL Taqman master mix (Applied Biosystems) and probes for RNU6B (assay ID: 001093), U6snRNA (assay ID: 001973), MiR-29a (assay ID: 002112), MiR-99a (assay ID: 000435), MiR-27b (assay ID: 000409), MiR-328-3p (assay ID: 000543), MiR-342-3p (assay ID: 002260), MiR-320a (assay ID: 002277), MiR-155 (assay ID: 002287) and MiR-34a (assay ID: 002316) in a final volume of 20 µL. Ten candidate microRNAs were selected from a review of previously published studies and were also chosen based on using prior related experiments. The PCR protocol was applied as follows: incubation for 10 min at 95 °C, followed by 40 cycles of 10 s at 95 °C and 1 min at 60 °C. The Ct values for RT-qPCR were determined using the QuantStudio™ Design & Analysis Software (Applied Biosystems) and the single-threshold method. PCR reactions were performed in a triplicate and experiments with coefficients of variation greater than 5% or that displayed unusual amplification curves were excluded from further analysis. A no-template control (NTC) and no reverse transcription controls (No-RT) were also included.

### Selection of candidate gene and stability analysis

A two test was designed to select and validate reference miRNAs for DR with greater accuracy. In the initial screening, the expression of 10 miRNAs (RNU6B, U6snRNA, MiR-29a, MiR-99a, MiR-27b, MiR-328-3p, MiR-342-3p, MiR-320a, MiR-155 and MiR-34a) was detected in plasma samples of 10 subjects of each group.

No expression could be detected for RNU6B, miR-27b, miR-155 and miR-34a. MiR-29a and MiR-99a also showed a low expression and homogeneity over the samples. Then, expression levels of the four remaining candidate endogenous normalizers (U6snRNA, MiR-320a, MiR-328-3p and MiR-342-3p) without significant difference between DR, DM without DR and healthy group (fold change <1) were selected for validation in all collected samples, as most suitable candidate reference genes in DR. Their stability was analyzed by Reffinder^29^ and NormFinder^30^ softwares.

NormFinder is a Microsoft Excel-based application that uses a model-based approach to assign a stability value to each candidate normalizer, accounting for intra- and inter-group variation. Exponentially transformed data (2^−Ct^ value) were used as input data in the NormFinder software. The lower stability value, the higher stability of the candidate.

The second tool used in this study is the online-based tool RefFinder (http://leonxie.esy.es/RefFinder/). It comprises four different commonly used normalization tools, namely Bestkeeper^34^, comparative Delta Ct^38^, NormFinder^29^ and GeNorm^35^, working with different algorithms to evaluate the most stably expressed gene ou gene pair of a specific sample set.

Bestkeeper^34^ analysis indicates the most stably expressed gene using the Pearson coefficient (r) of the Bestkeeper Index. Standard deviation is calculated based on the raw Ct values of each sample and high standard deviations are pointed as inadequate. GeNorm^35^ analysis analyzes the stability of candidate reference genes evaluating the average pairwise variation of a gene compared with all other genes, identifying the optimal number of reference genes required by analyzing the pairwise variation among candidate genes. The lowest M value indicates the most stable expression. The overall stability ranking of candidate genes was determined using the geometric mean of the rankings generated from all four analyses.

### Effect of normalization

In an independent experiment, we tested the effect of using MiR-328-3p alone and the average of all miRNAs displaying Ct < 32 cycles as showed in previous studies^31^ on relative expression values using MiR-342-3p as target to verify the effect of candidate reference genes on the accuracy of RT-qPCR results. Fold change of miRNAs were calculated using the 2^−ΔCT^ method. The ANOVA Analysis of variance test and post-hoc Tukey test were used to determine statistically significant differences in expression levels between DR, DM without DR and control groups. Statistical analysis was performed with GraphPad Prism 5.0 (GraphPad Software). P-values < 0.05 were considered statistically significant. All data generated or analyzed during this study are included in this published article.

## Data Availability

The data that support the findings of this study are available from the corresponding author, upon reasonable request.
